# The microRNA regulatory landscape of MSC-derived exosomes: a systems view

**DOI:** 10.1038/s41598-018-19581-x

**Published:** 2018-01-23

**Authors:** Scott W. Ferguson, Jinli Wang, Christine J. Lee, Maixian Liu, Sriram Neelamegham, John M. Canty, Juliane Nguyen

**Affiliations:** 1 Department of Pharmaceutical Sciences, School of Pharmacy, University at Buffalo, The State University of New York, Buffalo, NY 14214 USA; 2 Department of Chemical and Biological Engineering, Department of Biomedical Engineering, Clinical and Translational Research Center of the University at Buffalo, The State University of New York, Buffalo, NY 14260 USA; 3 Department of Medicine, Department of Physiology and Biophysics, Department of Biomedical Engineering, The Clinical and Translational Research Center of the University at Buffalo, Buffalo, New York and the VA Western New York Healthcare System, Buffalo, NY 14214 USA

## Abstract

Mesenchymal stem cell (MSC)-derived exosomes mediate tissue regeneration in a variety of diseases including ischemic heart injury, liver fibrosis, and cerebrovascular disease. Despite an increasing number of studies reporting the therapeutic effects of MSC exosomes, the underlying molecular mechanisms and their miRNA complement are poorly characterized. Here we microRNA (miRNA)-profiled MSC exosomes and conducted a network analysis to identify the dominant biological processes and pathways modulated by exosomal miRNAs. At a system level, miRNA-targeted genes were enriched for (cardio)vascular and angiogenesis processes in line with observed cardiovascular regenerative effects. Targeted pathways were related to Wnt signaling, pro-fibrotic signaling via TGF-β and PDGF, proliferation, and apoptosis. When tested, MSC exosomes reduced collagen production by cardiac fibroblasts, protected cardiomyocytes from apoptosis, and increased angiogenesis in HUVECs. The intrinsic beneficial effects were further improved by virus-free enrichment of MSC exosomes with network-informed regenerative miRNAs capable of promoting angiogenesis and cardiomyocyte proliferation. The data presented here help define the miRNA landscape of MSC exosomes, establish their biological functions through network analyses at a system level, and provide a platform for modulating the overall phenotypic effects of exosomes.

## Introduction

Exosomes are 50–100 nm lipid vesicles produced by multi-vesicular bodies (MVBs) prior to extracellular secretion^1,2^. Initially assumed to house unwanted ‘junk’ RNA, it is now thought that cells selectively sort specific non-coding (nc)RNAs into exosomes for active transport to neighboring cells^1,3^. Exosomes exert specific effects on their microenvironment and in doing so play important roles in intercellular communication in both healthy and diseased tissues^1,4^. For instance, exosomes secreted by mesenchymal stem cells (MSCs) can mediate the regeneration of injured and damaged tissues^5,6^.

MSC exosomes carry trophic factors through their protein, ncRNA, RNA, and lipid cargoes^7^. In the heart, they mediate cardiac tissue repair through various mechanisms including modulating the injured tissue environment, inducing angiogenesis, promoting proliferation, and preventing apoptosis^8,9^. Administration of MSC-derived exosomes after myocardial infarction (MI) reduces fibrosis^10^. Moreover, the regenerative effects of MSC exosomes are not limited to cardiac regeneration, and they have been shown to have beneficial effects in resolving acute lung injury, promoting neurite outgrowth after cerebral infarction, and inducing liver regeneration *in vivo*^5^. For instance, MSC exosomes have been shown to shorten the wound healing time and reduce scar formation by modulating cellular migration, proliferation, and collagen synthesis^11^. The precise mechanism regulating these beneficial effects remains unknown.

One of the most attractive features of exosomes is their ability to transfer cargo to recipient cells and in doing so modify cellular phenotypes^12^. microRNAs (miRNAs), which regulate the expression of approximately 30–70% of human genes through base-pairing of their “seed” sequences with complementary mRNA^13^, make up an important fraction of exosomal content. Thus, it is not surprising that miRNAs are key contributors to the overall biological function of exosomes. However, MSC exosomes contain diverse and numerous miRNAs, making it difficult to establish the contributory effects of individual miRNAs to the overall phenotypic response. Most previous studies have taken a candidate approach and singled out specific miRNAs to assess their therapeutic effects^14^, but this approach may not fully capture the various biological effects that miRNAs contained within MSC exosomes induce in recipient cells. A systems level analysis is warranted.

Here, we profiled and quantified the miRNA landscape in MSC exosomes and used a bioinformatics approach to identify which pathways and networks are most likely to be affected by exosomal miRNAs at a system level. The predicted regenerative effects of MSC exosomes were assessed using angiogenesis, cellular proliferation, and fibrosis assays. We then determined if the regenerative effects of MSC-derived exosomes could be further improved by enrichment with specific proliferation- and angiogenesis-promoting miRNAs. Our non-viral miRNA loading strategy significantly enriched miRNAs into exosomes to potentiate exosome-mediated phenotypes.

## Results and Discussion

### MSC exosomes contain numerous miRNAs predicted to regulate a tissue-regenerative target mRNA landscape

**Figure 1:**
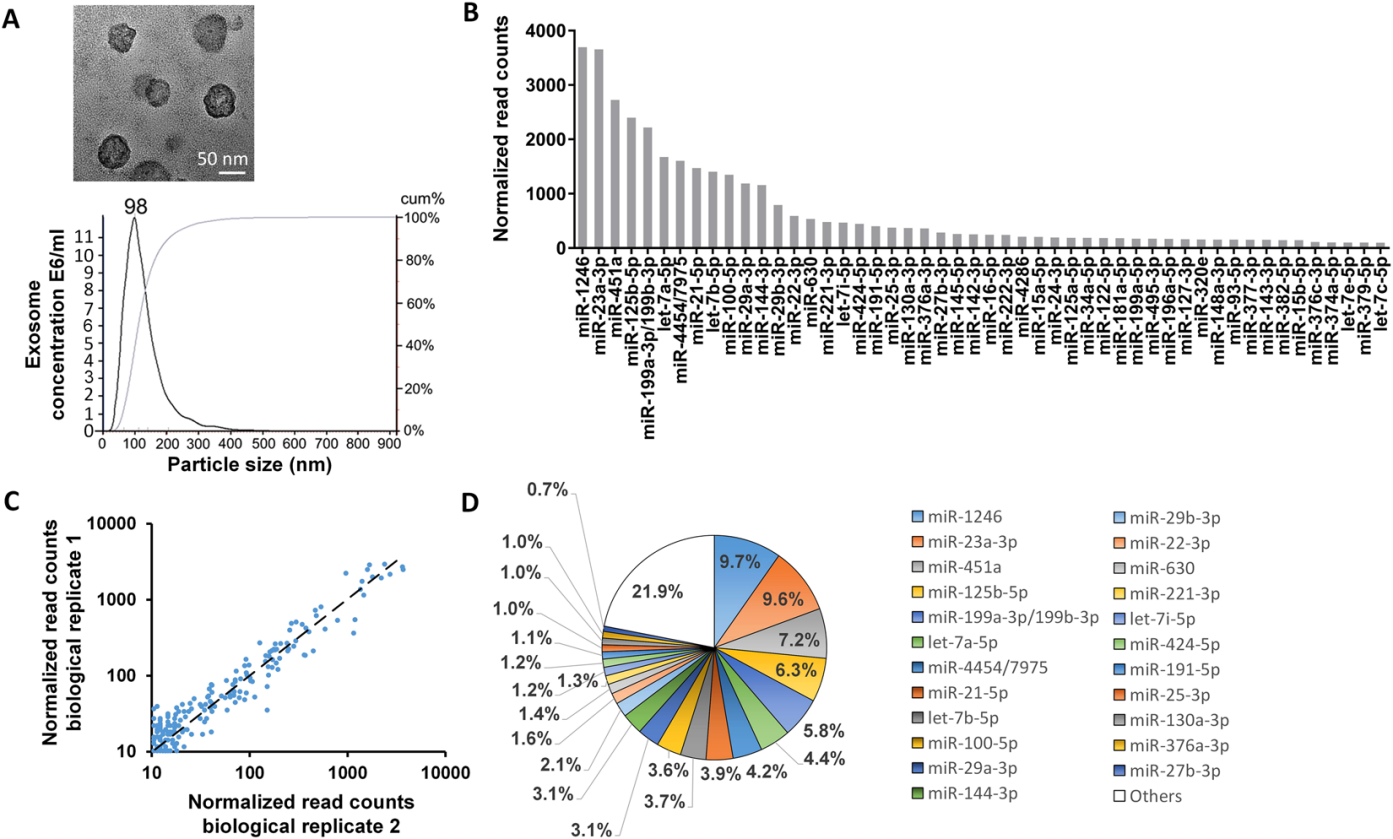
(**A**) TEM image of MSC exosomes and analysis of exosome size by nanoparticle tracking analysis. (**B**) Total reads of the top 50 miRNAs are shown. All miRNAs were normalized to positive and negative controls. miRNA profiling was performed using the NanoString platform and analyzed with nSolver Software 3. (**C**) Correlation of biological replicates of miRNA read counts of MSC exosomes. The Pearson correlation coefficient is 0.93. (**D**) The top 23 miRNAs account for 79.1% of total miRNAs present in MSC exosomes.

Mesenchymal stem cell (MSC)-secreted exosomes have been shown to regenerate tissues via their miRNA and protein cargoes^5,9,10^. Most studies have focused on single candidate exosomal proteins or RNAs, which although yielding important data do not take the overall effects of the entire exosomal cargo into account. Here we used miRNA profiling and bioinformatics to determine the miRNA landscape of MSC exosomes. After confirming that the exosomes isolated from MSCs were positive for the common exosomal marker CD63 (Supplementary Fig. 1), the size of exosomes were analyzed by nanoparticle tracking analysis (NTA) and transmission electron microscopy (TEM). The hydrodynamic diameter of exosomes measured by nanoparticle tracking analysis (NTA) was ~98 nm (Fig. 1A). Exosomes were 55.5 ± 11.1 nm in diameter when measured by TEM (100 exosomes were counted). A representative image is shown in Fig. 1A. The difference in size could be due to the fact that exosomes were in their natural, solvated state when measured by NTA while they were dry under TEM conditions. Studies have shown that the method by which exosomes are dried or desiccated can affect their size and thus can differ from hydrated exosomes^15^.

MSC miRNAs were profiled by NanoString and rank ordered according to total reads (highest to lowest; Fig. 1B) normalized to positive controls and reference genes; read counts were highly correlated between biological replicates (Fig. 1C). The top 23 miRNAs accounted for 79.1% of all the exosomal miRNA content (Fig. 1D); since the remaining 148 miRNAs were present at very low read counts and made up a very small percentage of the total reads (0.03% to 0.7%), they were deemed unlikely to have significant biological effects compared to more abundant miRNAs, and thus were excluded from further analysis. The average read count of the top 23 miRNAs were at least 22.3 times more abundant than the average read count of the remaining MSC miRNAs (Supplementary File – Excel Table 1).

**Figure 2:**
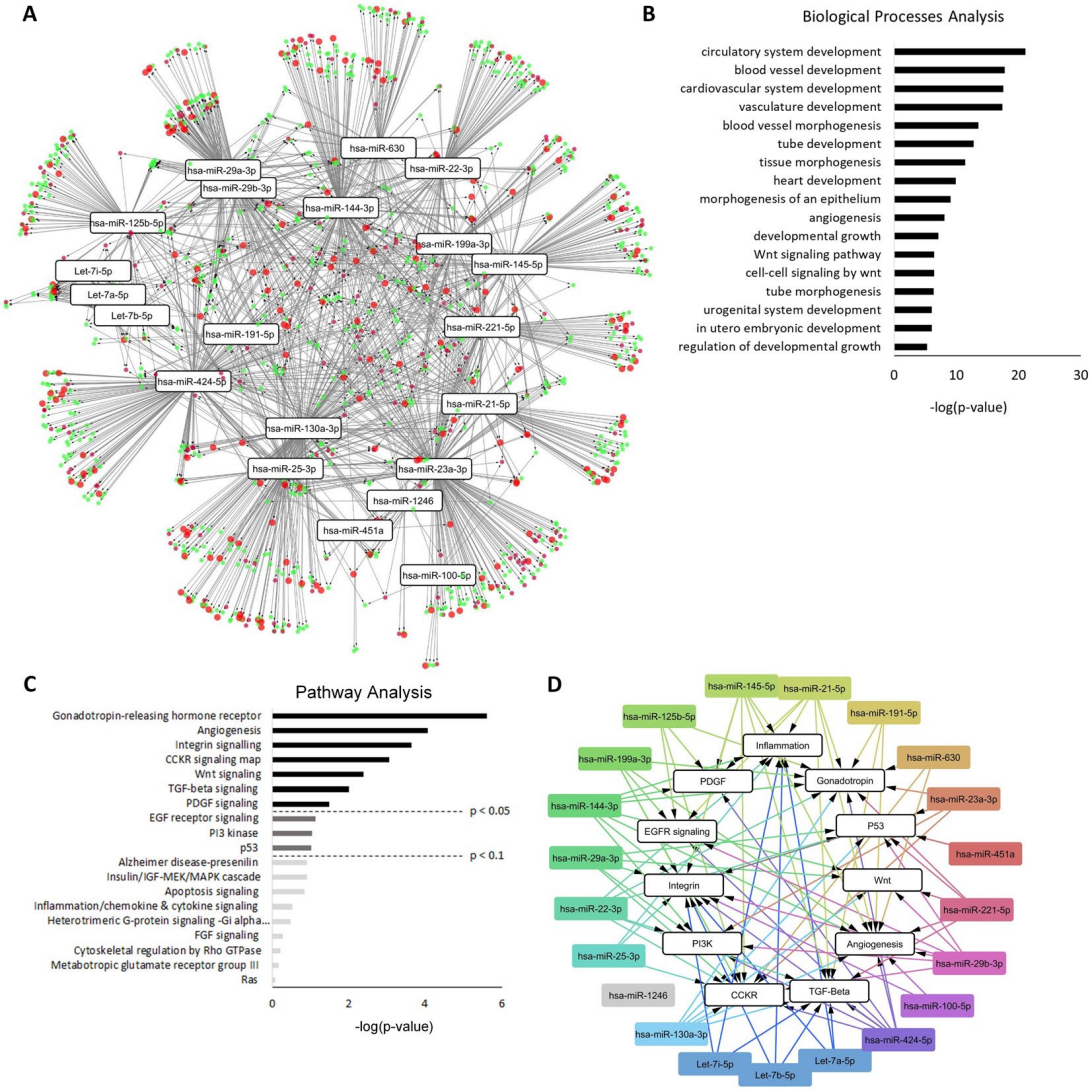
Processes and pathways targeted by the most abundant miRNAs in MSC exosomes. The 23 most abundant miRNAs contained within MSC exosomes were analyzed using miRDIP to determine the target gene landscape (5,481 genes). Of these, 1,317 were in the top two-thirds in confidence and were further analyzed by PANTHER to identify statistically enriched biological processes and pathways regulated by the target genes. Only GO terms enriched more than one variance above the mean (1.81-fold) and meeting statistical significance (p < 1e-6) were considered likely biological processes and pathways. **(A)** Enrichment map of biological processes targeted by the top 23 miRNAs in MSC exosomes. Nodes represent the individual genes of significantly enriched biological processes and the miRNAs which target them, connected by the edges. Genes related to the biological processes of vascularization are shown as small red circles, growth-related genes are shown in green circles, and genes with overlapping contributions to vascularization and growth are shown as large red circles. miRNAs targeting similar gene clusters are located in close proximity to one another (e.g., let-7(a,b,i)-5p and hsa-miR-29(a,b)-3p). (**B**) Enriched biological processes and (**C**) enriched pathways targeted by the top 23 miRNAs ranked based on significance from high to low. (**D**) Enrichment map of pathways targeted by the top 23 miRNAs. Nodes represent significantly enriched pathways and the miRNAs which target them.

Collectively, the top 23 miRNAs were predicted to target 5,481 genes with high stringency (above the bottom third in confidence, present in at least five databases as curated by miRDIP), a breakdown of which is shown in Supplementary Fig. 2. The miRNA target geneset was analyzed by PANTHER to identify statistically over-represented pathways and biological processes (Fig. 2**)**; the majority of the genes substantially contributed to specific processes related to (a) cardiovascular development, angiogenesis, and tube formation, (b) pathways related to cell death and growth, and (c) pathways related to fibrosis such as Wnt signaling, PDGF, and TGF-β. The top miRNAs affecting the most targets with respect to circulatory system development, comprised of processes related to vasculature and tube development, were miR-23a-3p, miR-424-5p, miR-144, and miR-130a-3p (>90 targets); between 9 and 85 cardiovascular development, angiogenesis, and tube formation genes were targeted by the remaining miRNAs. miR-23a-3p, miR-424-5p, miR-144-3p, miR-130-3p, miR-145-5p, miR-29b-3p, miR-29a-3p, miR-25-3p, miR-221-5p, miR-21-5p, miR-125b-5p, miR-22-3p, miR-199a-3p, and miR-191-5p affected over 60 genes implicated in the regulation of cell growth, with the remaining miRNAs affecting less than 29 genes associated with these pathways (Supplementary Fig. 2A,B).

### Angiogenesis and tissue remodeling pathways dominate the MSC exosome miRNA target landscape

To gain a better understanding of how the genes, pathways, and biological processes regulated by the top 23 miRNAs relate to one another, the complex network of miRNAs, biological processes, and pathways was visualized in Cytoscape. As shown in Fig. 2A, the top 23 miRNAs were predicted to mainly target genes controlling processes related to vascularization and cellular growth (green, red, and large red circles). Many of the miRNAs such as let-7(a,b,i) and miR-29(a,b)-3p target similar gene clusters, as shown by their close proximity when visualized in Cytoscape (Fig. 2A). A detailed breakdown of the biological processes showed that these miRNAs target genes that regulate circulatory, blood vessel, cardiovascular system, and vascular development and angiogenesis (Fig. 2B).

When miRNA target genes were analyzed with regard to pathways (Fig. 2C), angiogenesis and integrin signaling pathways dominated, which control cell mobility and migration as well as vascular and tube formation^16^. Other targeted pathways included Wnt signaling, TGF-β signaling, and platelet-derived growth factor (PDGF) signaling, which are implicated in the modulation of fibrosis^17–20^. The TGF-β signaling pathway promotes extracellular matrix deposition by increasing collagen and fibronectin synthesis and by modulating the fibroblast phenotype^17^. Similar to TGF-β, PDGF has been shown to mediate pro-fibrotic effects by inducing extracellular matrix synthesis and fibronectin, collagen, and proteoglycan expression^19^. Several miRNAs have been attributed with anti-fibrotic activity in the heart^21^, some of which are present within the top 23 enriched miRNAs in MSC exosomes. Among the most prominent are the miR-29 family and Let-7i^22,23^.

In our network analysis these miRNAs are predicted to target the TGF-β pathway. These miRNAs directly target *COL1A1* and between the miR-29 and the Let-7 family miRNAs in the top 23, 13 other isoforms of collagen are also targeted. Additionally miR-29a and miR-29b target *FBN1*; it is clear that these miRNA provide substantial coverage against a host of fibrotic genes. Additional pathways that showed a trend towards enrichment involved processes related to cell growth, proliferation, and apoptosis and included cell epidermal growth factor receptor (EGFR) signaling and the PI3 kinase and p53 pathways (p = 0.07–0.09).

### Bulk MSC exosomes can modify fibrosis and angiogenesis *in vitro*

**Figure 3:**
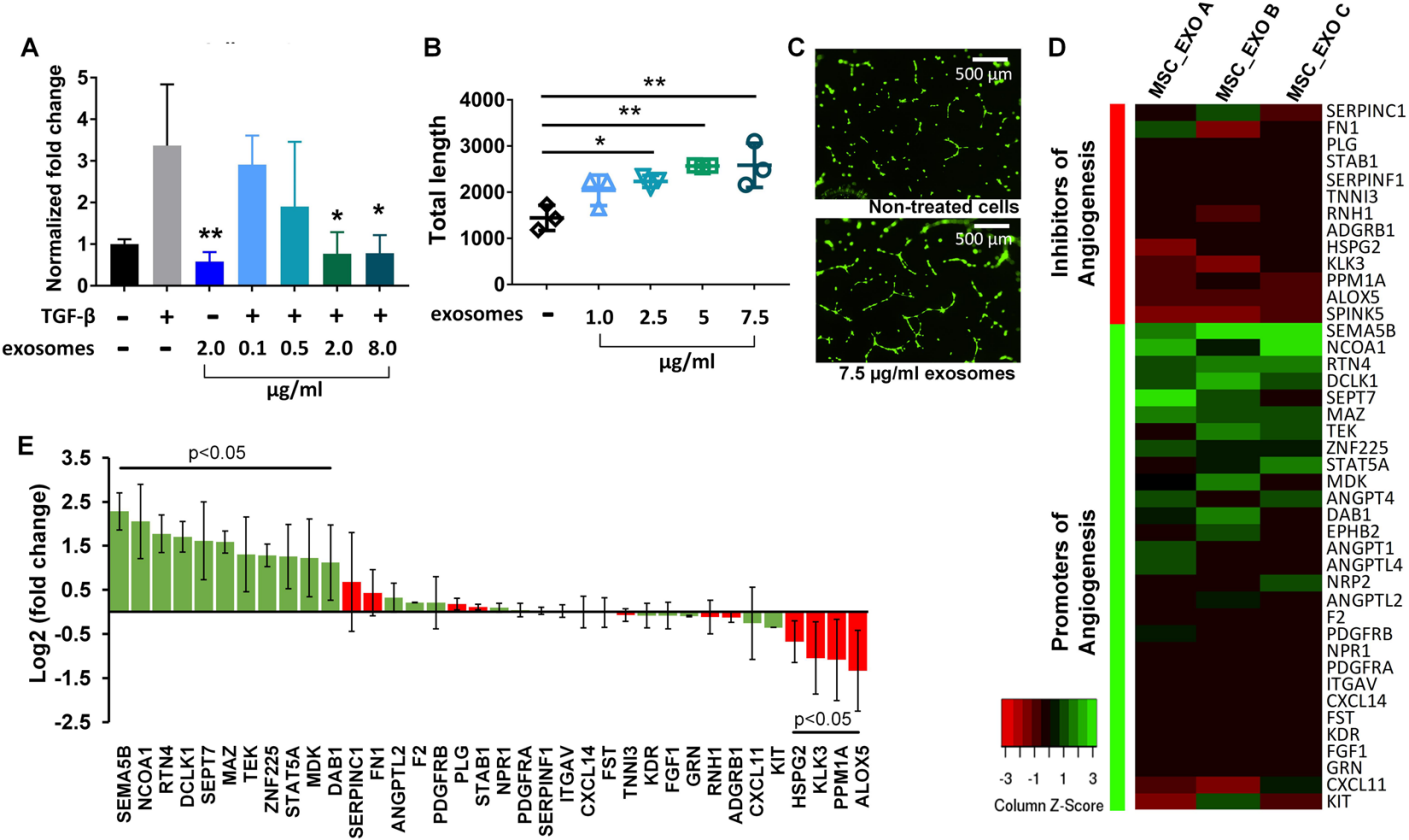
(**A**) Exosomes inhibit collagen I gene production in primary human cardiac fibroblasts. Human cardiac fibroblasts were serum-starved for 12 hours in DMEM with or without MSC exosomes (0.1, 0.5, 2.0 and 8.0 μg). 10 ng of TGF- β in 500 μl of complete media was then added to stimulate fibrosis. Non-treated cells served as controls (−/−). Collagen I mRNA levels were normalized to GAPDH. (**B**,**C**) Unmodified exosomes increased the total length of segments, isolated elements, and branches in HUVECs in a dose-dependent manner. HUVECs were stained with Calcein. (**D**) Heat map showing upregulated and downregulated genes when HUVECs were treated with MSC exosomes (n = 3). (**E**) Fold-enrichment of pro-apoptotic and anti-apoptotic genes in HUVECs treated with MSC exosomes compared to non-treated control cells (n = 3). Fold-changes and statistical analysis for (**D**,**E**) were performed using DEseq 2.

Given the predicted effects of the MSC miRNA target landscape on fibrosis, angiogenesis, and potentially cell growth, we next assessed the *in silico-*predicted biological effects of MSC exosomes in three representative assays. As shown in Fig. 3A, incubation of human cardiac fibroblasts with MSC exosomes inhibited collagen I production in a dose-dependent manner in TGF-β-stimulated cells and reversed collagen levels back to baseline (p < 0.05), consistent with reports of the anti-fibrotic effects of exosomes when administered to animal models with induced myocardial infarction^24^. Collagen I production plays an integral role in fibrosis and is regulated through Wnt, PDGF, and TGF-β signaling pathways, predicted targets of the exosomal miRNA landscape^18,25^.

According to our bioinformatics analyses, numerous genes targeted by MSC-derived exosomal miRNAs were related to angiogenesis and vascular development. We therefore performed an angiogenesis assay and incubated human umbilical vein endothelial cells (HUVECs) plated onto Geltrex with increasing concentrations of unmodified exosomes. MSC exosomes were taken up into HUVECs in a time-dependent manner (Supplementary Fig. 3). As shown in Fig. 3B,C, at 2.5 ug/ml concentrations and above, unmodified exosomes significantly increased the total lengths of segments, isolated elements, and branches (abbreviated as total lengths) in the angiogenesis assay, consistent with published studies showing that MSC exosomes can induce angiogenesis in various animal models^26,27^. Several of the top 23 miRNAs have previously been shown to mediate angiogenesis. For example, miR-21 has been shown to induce angiogenesis through activation of protein kinase B (AKT) and the extracellular signal-regulated kinase (ERK), with overexpression in DU145 cells increasing expression of VEGF and hypoxia-inducible factor 1 (HIF1)-alpha and consequent angiogenesis^14^. miR-1246 is also implicated in angiogenesis by activating Smad 1/5/8 signaling in HUVECs. miR-1246 then directly targets *PML* to inactivate Smad 2/3 signaling, leading to compensatory activation of Smad 1/5/8 signaling and angiogenesis^28^. miR23a-3p belongs to a miRNA cluster (miR-23∼27∼24 gene clusters) expressed in endothelial cells and highly vascularized organs such as the lung and heart. miR-23 has been shown to enhance angiogenesis by targeting *Sprouty2* and *Sema6A*, and miR-23 knockdown inhibited the proliferation and migration of endothelial cells in the presence of VEGF^29^.

While angiogenic effects were reported for individual miRNAs in the top 23, the mechanisms vary and it is still unclear which specific genes are targeted by the exosomal MSC miRNAs. In order to examine the mechanisms of MSC exosomes in promoting angiogenesis we performed mRNA sequencing (Illumina) on HUVECs treated with MSC exosomes (Fig. 3D,E**)**. The mRNA sequencing analyses revealed that a number of genes promoting angiogenesis were upregulated more than 2-fold compared to non-treated cells. Some genes were not detected in any control samples, but were detected in the MSC exosome treated samples. These include numerous angiopoietin genes: ANGPT1, ANGPT4, and ANGPTL4^30^ as well as other important mediators of angiogenesis such as ephrin type-B receptor 2 (EPHB2), and neuropilin 2 (NRP2)^31,32^. Further, the angiopoietin receptor, TEK tyrosine kinase, was also upregulated (2.5-fold), indicating the angiopoietin network may be a primary target of MSC-exosomes in promotion of angiogenesis^33^. Also upregulated were MYC-associated zinc finger protein (MAZ) (3.0-fold), semaphorin 5B (SEMA5B) (4.9-fold) and, nuclear receptor coactivator 1 (NCOA1) (4.2-fold) all of which are either correlated with or known to increase the expression of VEGF^34–36^. Downstream of VEGF, the reelin adaptor protein (DAB1) gene was also upregulated (2.2-fold)^37^. The transcription factors STAT5A (2.4-fold) and ZNF225/EGR1 (2.4-fold) mediate their angiogenic effects through FGF^38,39^, while reticulon 4 (RTN4/NOGO) (3.4-fold) signals through protein kinase B (Akt) similarly to VEGF to promote angiogenesis^40,41^. A number of the γ-secretase complex genes were regulated by MSC exosomes including presenilin enhancer gamma-secretase subunit (PSENEN) (5.2-fold) which could influence angiogenesis dynamics through the NOTCH pathway^42,43^.

Additionally, several genes that are known to inhibit angiogenesis were downregulated in HUVECs treated with MSC exosomes. Serine protease inhibitor Kazal-type 5 (SPINK5), a negative regulator of angiogenesis, was detected in the control samples, but was not detected in any of the MSC exosome treated samples^44^. Arachidonate 5-Lipoxygenase (ALOX5) (−3.3-fold) and protein phosphatase 1 A (PPM1A) (−2.8-fold) were significantly downregulated and are also reported as negative regulators of angiogenesis (Fig. 3D,E**)**^45,46^.

### Effects of MSC exosomes on cardiomyocyte apoptosis and proliferation

**Figure 4:**
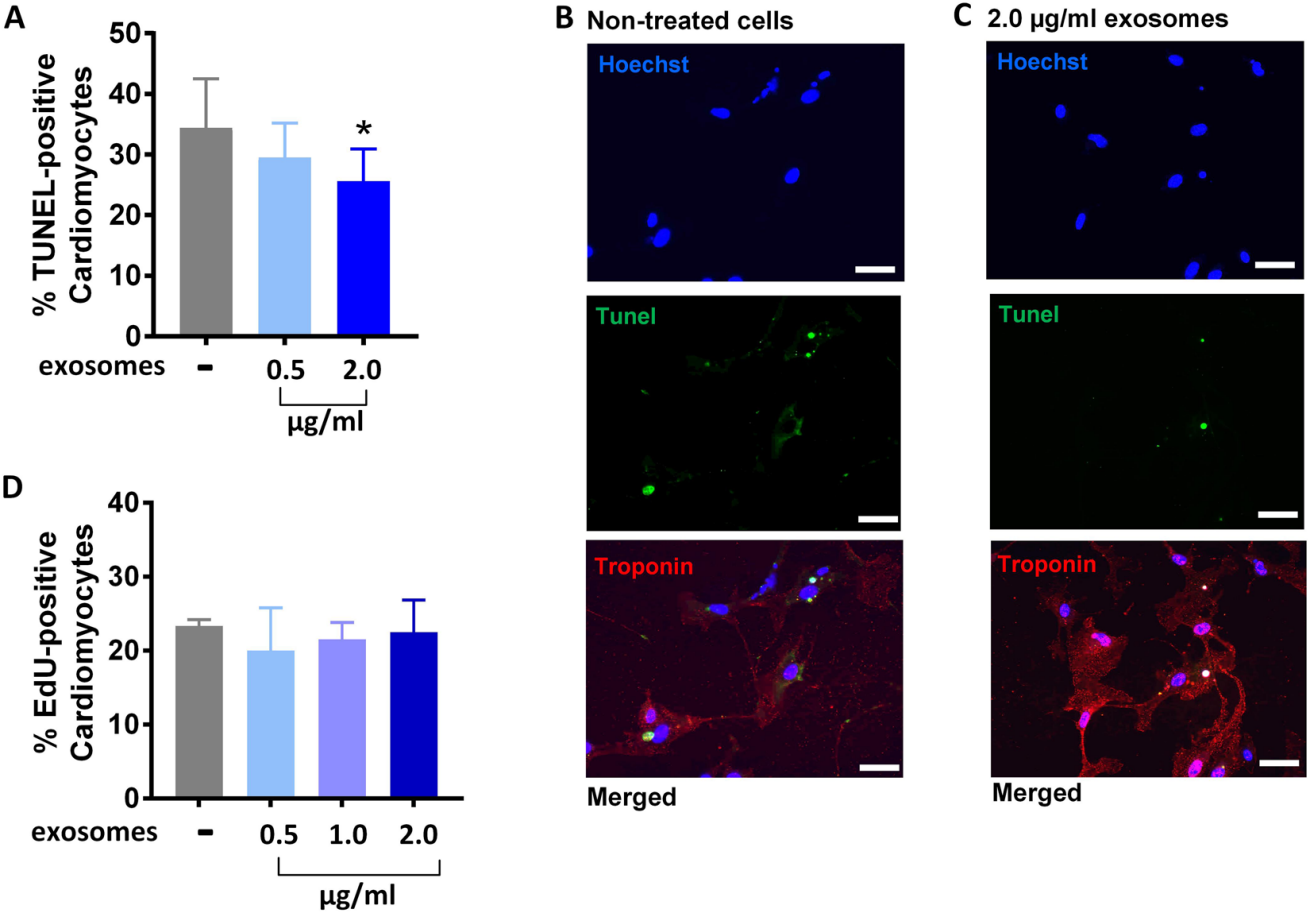
**(A,B,C)** Effects of unmodified MSC exosomes on cardiomyocyte apoptosis. **(B,C)** Nuclei were labeled with Hoechst (blue), TUNEL-positive nuclei were labeled with Alexa 488 (green), and cardiomyocytes were stained with Troponin T (red). Scale bar: 50 µm. (**D**) Effect of unmodified MSC exosomes on cardiomyocyte proliferation. Data are mean ± sd, by one-way ANOVA with Tukey post-test (*p < 0.05, **p < 0.01).

Our bioinformatic analysis provided strong evidence that MSC exosomes would modulate fibrosis and angiogenesis via their miRNA cargo, which was confirmed *in vitro*. We next sought to examine whether MSC exosomes were also able to show efficacy in enriched but not statistically significant pathways such as inhibiting cardiomyocyte apoptosis (Fig. 4A–C) as suggested by p53 pathway representation in MSC exosome miRNA targets. Notably, unmodified exosomes were able to significantly inhibit cardiomyocyte apoptosis at higher doses (2 ug/ml, p < 0.05). p53 is intimately involved in apoptosis in a variety of mammalian cells including cardiomyocytes^47^. In models of myocardial infarction, increased p53 levels are correlated with enhanced cardiomyocyte apoptosis^48,49^. Conversely, it has been shown that allografts from p53 deficient mice display lower cardiomyocyte apoptosis than allografts from p53 competent mice^50^. Aside from p53-mediated apoptosis, other pathways might be responsible for inducing cell death in cardiomyocytes.

Because our network analysis showed a positive trend towards significance in pathways related to growth and proliferation (EGF-pathway, PI-3K, and p53) (p = 0.07 to p = 0.09), we next tested whether MSC exosomes were able to induce cardiomyocyte proliferation. Although three of the top 23 miRNAs (miR-199a, miR-424-5p, and miR-21-5p) target Crim1 (Supplementary File - Excel Table 2), a gene that is instrumental in blocking cardiomyocyte proliferation^51^, at the concentrations tested (2 µg/ml exosomes), cardiomyocytes did not show enhanced proliferation when incubated with MSC exosomes (Fig. 4D).

To assess if the phenotypic effects of exosomes can be modulated by enrichment with specific miRNAs, we next developed a loading method that allows enrichment of exosomes with nucleic acids.

### Enhancing exosomal function with specific miRNA cargoes

**Figure 5:**
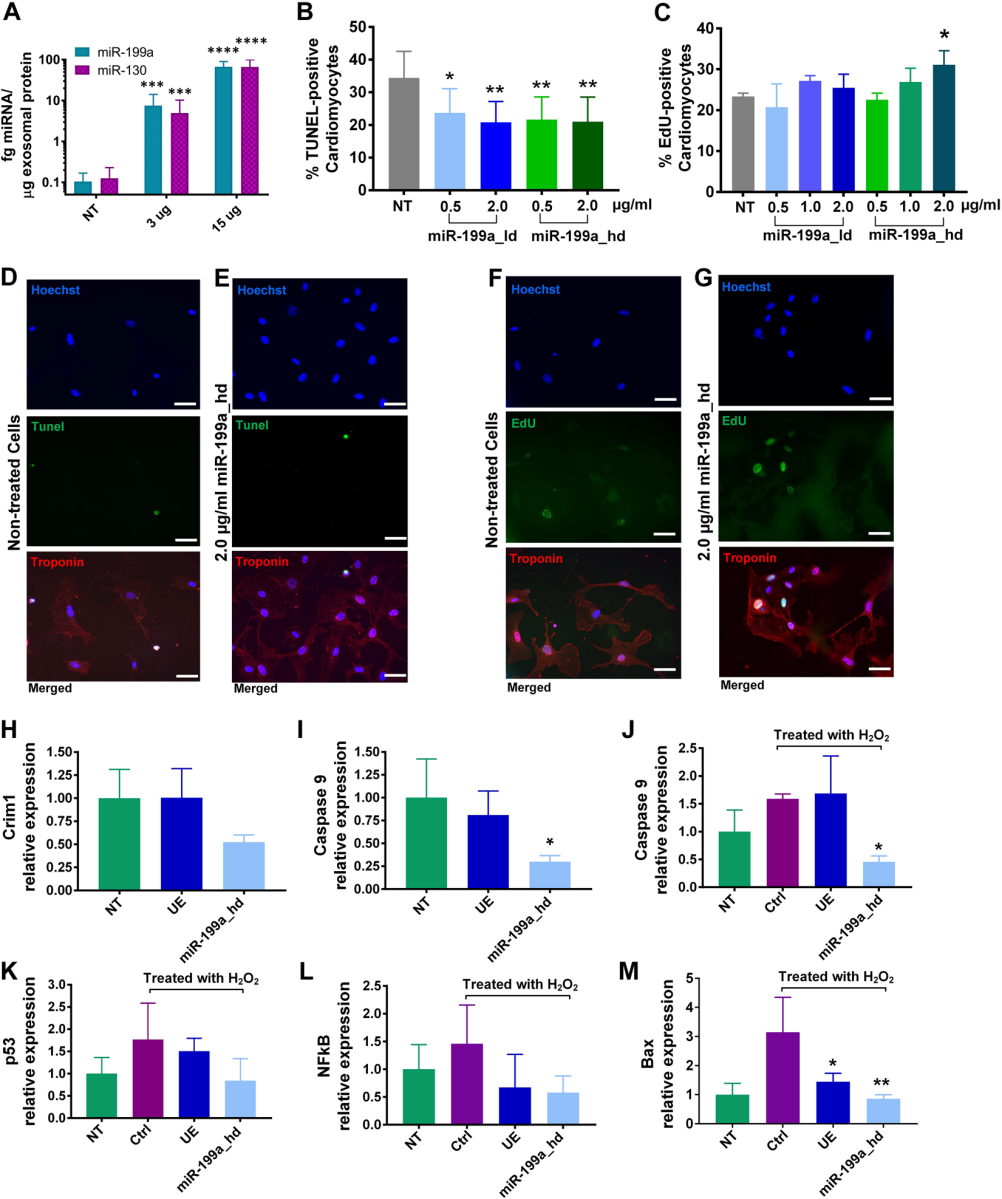
**(A)** Loading of miR-199a-3p and miR-130 into MSC exosomes. (**B,D,E**) Effects of miR-199a-3p loaded exosomes on cardiomyocyte apoptosis. **(C,F,G)** Effects of miR-199a-3p loaded exosomes on cardiomyocyte proliferation. **(D,E)** Nuclei were labeled with Hoechst (blue), TUNEL-positive nuclei were labeled with Alexa Fluor 488 (green), cardiomyocytes were stained with Troponin T (red). Scale bar = 50 µm. **(F,G)** Nuclei were labeled with Hoechst (blue), newly proliferated nuclei were labeled with EDU (green), and cardiomyocytes were stained with Troponin T (red). Scale bar = 50 µm. (**H,I**) miR-199a-3p promotes cardiomyocyte proliferation through Crim1 and Caspase 9 downregulation. **(J,K,L,M)** Expression of apoptosis markers in hydrogen peroxide treated cardiomyocytes following pretreatment with 2 µg/ml of MSC exosomes or 2 µg/ml of miR-199a-loaded MSC exosomes. n = 3 for **A,B,C,H,I**; n = 4 for **J,K,L,M**. Data are presented as mean ± SD, with *p < 0.05 and **p < 0.01 by one-way ANOVA with Tukey post-test. NT = non-treated cells, ctrl = control, UE = unmodified exosomes, miR-199a_hd = exosomes loaded with high dose of miR-199a.

To engineer cargo-enriched MSC exosomes, we developed a virus-free miRNA loading approach suitable for *ex vivo* manipulation of many cell types including primary cells and difficult to transfect cell lines. A virus-free approach has advantages for clinical application since it poses fewer risks to patients. Although cationic transfection reagents have previously been used, they can lead to the undesired incorporation of the transfection reagents into exosomes^52^. Direct electroporation of the exosome-producing cells does not require viral or chemical reagents and allows miRNA deposition into the cytoplasm for direct incorporation into exosomes. miRNA loading into exosomes was dose dependent, as shown in Fig. 5A. A 520- to 640-fold enrichment of miRNAs into exosomes was observed using the virus-free electroporation approach.

We first selected miR-199a for further analysis, because it has been shown to induce proliferation in cardiomyocytes, an endpoint which we were unable to achieve with unmodified MSC exosomes. Eulalio and colleagues found that miR-199a targets *Crim1* and increased cardiomyocyte proliferation by 30% in a functional high-throughput screen^51^. Further, based on our gene target predictions of exosomal MSC miRNAs using mirDIP, miR-199a targeted 22 genes implicated in cell death/proliferation and cell cycle regulation that were not targeted by any other of the top 23 miRNAs found in MSC exosomes (Supplementary Table 3) including *RB1*, which leads to cell cycle arrest^53^, *LKB1*, specific loss of which results in cellular proliferation^54^, and *NEUROD1*, a gene present in terminally differentiated tissues that when overexpressed leads to cell cycle arrest^55^. This highlights the important role of miR-199a in regulating cellular proliferation. As shown in Fig. 5C, miR-199a loaded exosomes increased cardiomyocyte proliferation in a dose-dependent manner with exosomes loaded with a high dose of miR-199a (miR-199a_hd) showing the highest effects. The proliferation of cardiomyocytes increased from a baseline level of 22% to ~30% when incubated with 2 ug of MSC exosomes loaded with high doses of miR-199a. Expression of Crim1 was not altered by the addition of unmodified MSC exosomes, but was reduced to 52% of control when cardiomyocytes were treated with miR-199a-3p loaded exosomes **(**Fig. 5H**)**.

Further, miR-199a loaded exosomes significantly inhibited cardiomyocyte apoptosis as shown in the TUNEL assay (Fig. 5B**)**. While 2 µg of unmodified MSC exosomes were required to reduce TUNEL-positive cardiomyocytes from ~34% to ~25%, miR-199a loaded exosomes were able to decrease TUNEL-positive cardiomyocytes down to ~21% at a 4-fold lower concentration of exosomes (0.5 µg). In order to understand the mechanism of action of MSC exosomes and miR-199a-3p loaded exosomes in preventing cardiomyocyte apoptosis cardiomyocytes were treated with hydrogen peroxide to induce cell death. We then analyzed the expression levels of apoptotic markers (p53, NFkB, BAX, and caspase 9) in exosome treated and non-treated cardiomyocytes. All apoptotic gene markers were upregulated by peroxide treatment (Fig. 5J–M). Treatment of apoptosis-induced cardiomyocytes with unmodified exosomes (UE) showed a trend towards BAX, p53, and NFkB reduction. The miR-199a-3p loaded exosomes significantly decreased BAX expression (p = 0.011) and mediated further reductions of p53, NFkB, and caspase 9. This is in line with a recent study that showed that miR-199a-3p mediates suppression of p53 expression through targeting of CABLES1^56^. CABLES1 is one of the predicted targets of miR-199a-3p (Supplementary Table 3). The increased potency of the miR-199a loaded exosomes is likely due to its ability to lower the expression levels of multiple apoptotic-inducing genes, including caspase 9, an important initiator in the apoptosis signaling cascade. The loaded exosomes reduced expression of caspase 9 below control cardiomyocytes that were not apoptosis-induced with hydrogen peroxide (p = 0.03).

Although MSC exosomes showed greater intrinsic potential for increasing angiogenesis than promoting cardiomyocyte proliferation, the angiogenic effects were relatively modest at the lower doses. As demonstrated by enriching exosomes with miR-199a, our loading approach has the potential to generate more potent exosomes that achieve prominent effects even at low exosome doses. To test whether the intrinsic angiogenic effects of MSC exosomes can be further improved, we chose miR-130a-3p.

**Figure 6:**
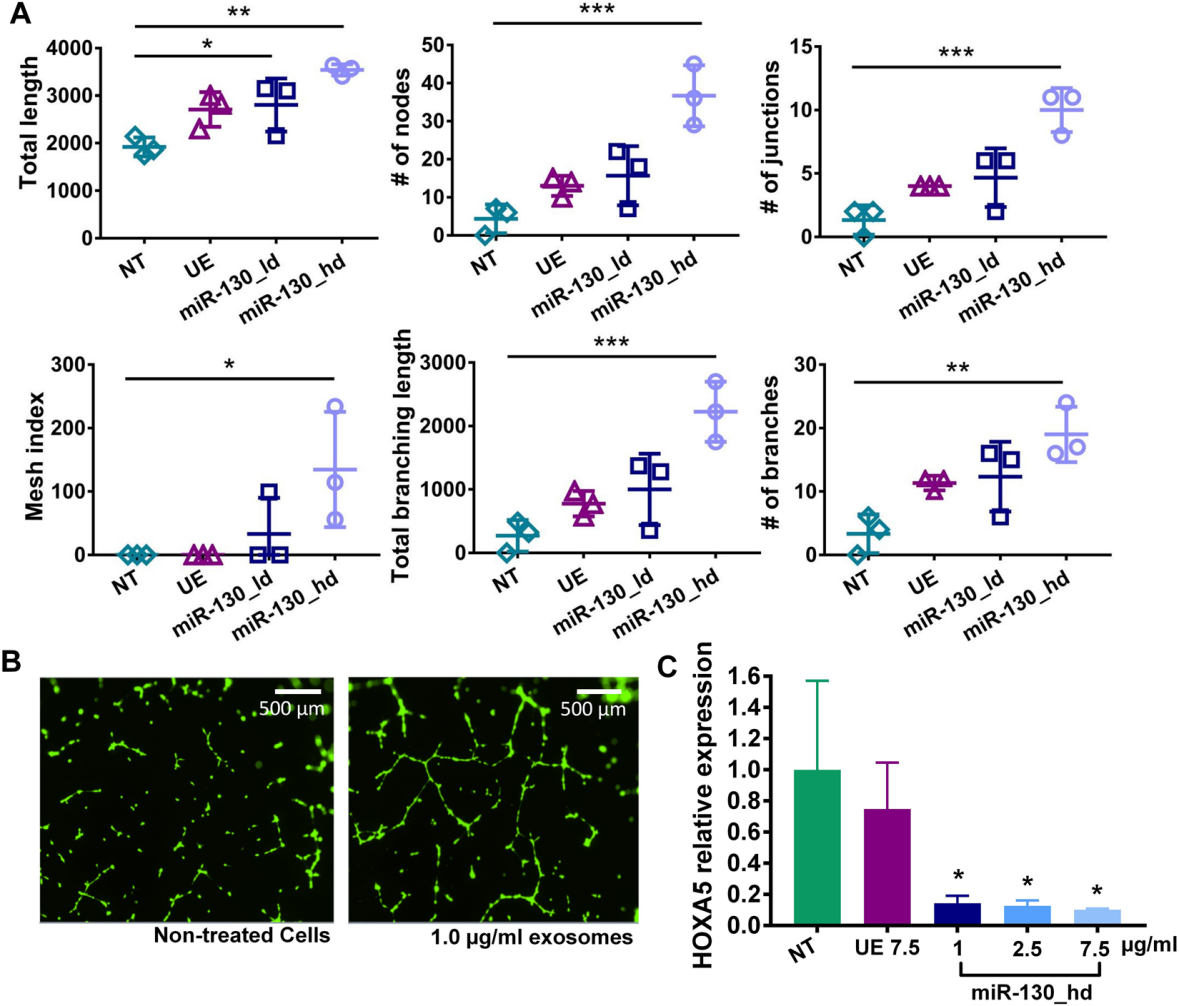
**(A & B)** Effects of miR-130a-3p-loaded exosomes on angiogenesis in HUVECs, data are presented as mean ± SD, n = 3 with *p < 0.05, **p < 0.01, ***p < 0.01 by one-way ANOVA with Tukey post-test. (**C**) miR-130a-3p promotes angiogenesis through downregulation of HOXA5. NT = non-treated cells, UE = unmodified exosomes, UE 7.5 = unmodified exosomes at 7.5 µg/ml, miR-130_hd = exosomes loaded with high dose of miR-130a-3p.

According to our network analysis, miR-23a-3p and miR-130a-3p target the most genes related to angiogenesis and vasculature development (104 and 99 genes, respectively) (Supplementary Fig. 5). Our analyses using mirDIP also showed that miR-130a-3p targets *HOXA5*, which when downregulated induces angiogenesis (Supplementary Fig. 5)^57^. Thus, we hypothesized that loading exosomes with miR-130a-3p would further enhance their angiogenic effects. MSC exosomes enriched with high doses of miR-130a-3p (miR-130_hd) increased all angiogenesis endpoints in a dose-dependent manner, with statistically significant differences at the highest dose (Fig. 6A–C). miR-130a-3p-loaded MSC exosomes achieved a greater than 1.5-fold increase in total length (total segments, isolated elements, and branches) and a 7-fold increase in the number of nodes and junctions and the establishment of a mesh index greater than 100. Non-treated or unmodified exosome-treated HUVECs did not score on the mesh index (Supplementary Fig. 6), indicating that a capillary-like network of tubular structures only emerged with specific enrichment with miR-130a-3p (Fig. 6A,B). The angiogenesis-inducing effects of miR-130a-3p have been shown to occur through the downregulation of antiangiogenic homeobox genes *GAX* and *HOXA5*^57^. We confirmed ~90% reduction in HOXA5 expression using miR-130a-3p loaded exosomes, which is in agreement with the established mechanism of action (p < 0.05) (Fig. 6C).

## Conclusions

MSC exosomes are being widely explored as a cell-free therapy in cardiac regeneration following myocardial infarction^58^. However, their biological function is not fully understood. Here we used network analyses to gain a comprehensive view of the main pathways and biological processes targeted by the most abundant miRNAs present in MSC exosomes. These included several processes and pathways involved in (cardio)vascular development, angiogenesis, cellular proliferation, and fibrosis. MSC exosomes mediate angiogenic, anti-fibrotic, and anti-apoptotic phenotypes and can be simply modified with specific cargoes using a virus-free approach to enhance their phenotypic effects. Enriching MSC exosomes with network-informed miRNA further improved on the intrinsic ability of MSC exosomes to prevent apoptosis, promote angiogenesis, and induce cardiomyocyte proliferation while making them more potent at lower concentrations compared to unmodified exosomes. These data help define the MSC miRNA landscape, establish their biological functions on a system level, and provide a platform for further improving their intrinsic regenerative effects in the search for clinically viable exosome-based therapeutics.

## Materials and Methods

### Exosome isolation

Human bone marrow-derived mesenchymal stem cells (MSCs) were obtained from the American Type Culture Collection (ATCC^®^ PCS-500-012) and cells between passage numbers P1-P7 were used for experiments. Exosomes were isolated from MSCs using the Total Exosome Isolation Reagent (Invitrogen, Life Technologies, Carlsbad, CA). 48 hours before exosome isolation MSCs were cultured in exosome-free media. To obtain exosomes, cell culture supernatants were harvested and centrifuged at 2000 × g for 30 min to remove cells and debris. Exosome isolation agent was added to the supernatant in a new tube prior to incubation overnight at 4 °C. The mixture was centrifuged at 10,000 × g for 1 h at 4 °C to pellet exosomes before re-suspension in PBS.

### Transmission electron microscopy (TEM)

Exosomes were prepared at a concentration of ~1 mg/mL. A 1% sample of uranyl acetate was prepared for negative staining. Type A carbon TEM grids (Ted Pella, Redding, CA) were placed on Whatmann filter paper (GE Healthcare, Maidstone, UK), and 20 μL of the sample was dropped onto the grid. The grid was left to air dry for 3 min. Immediately after, 20 μL of 1% uranyl acetate was added to the grid. After 3 min incubation, the remaining liquid was wicked away and the grids allowed to air dry. The grid was analyzed using a FEI Techni F20 G2 transmission electron microscope (FEI, Hillsboro, OR) at an accelerating voltage of 200 kV.

### miRNA profiling

Total RNA was extracted from exosomes, and miRNA profiling was performed using the NanoString platform (NanoString Technologies, Seattle, WA) according to the manufacturer’s instructions. nSolver was used to normalize the number of reads of each miRNA to positive controls and to the top 100 reads. Negative controls were used as a threshold for background subtraction. The Pearson correlation of two biological replicates were determined.

### miRNA pathway and network analyses

The top 23 miRNAs identified in MSC exosomes and predicted to be present at sufficient quantity to exert biological activity were analyzed using mirDIP, which integrates several miRNA target databases, to determine miRNA target genes with high confidence. For increased stringency, only miRNA targets present within five databases were considered (3,364 genes). Of these genes, 1,317 were in the top two-thirds in confidence and were further analyzed by PANTHER to identify enriched biological pathways. Genes were considered to be highly targeted by the 23 miRNAs if the gene ontology (GO) terms showed more than one variance of enrichment above the mean (1.81-fold) and if they met a statistical significance of p < 1e-6. For biological process-related GO terms, processes were only considered significantly targeted if they were enriched more than one standard deviation above the mean (1.73-fold) and had p-values < 1e-6.

### Tube formation assay

Primary human umbilical vein endothelial cells (HUVECs) were obtained from Gibco (Cat#: C0035C) and cultured in Medium 200 (Cat#: M200500, ThermoFisher Scientific) containing large vessel endothelial supplement (Catalog #: A1460801, ThermoFisher Scientific). HUVECs were incubated with 1 µg/ml, 2.5 µg/ml, 5 µg/ml, and 7.5 µg/ml of unmodified exosomes and 1 µg/ml of exosomes enriched with miR-130a-3p for 24 h. Then, 5,000 exosome-treated HUVECs were plated into wells coated with Geltrex (Cat#: A1413201, ThermoFisher Scientific). 17 h after plating, HUVECs were stained with calcein and imaged at em/ex 488/505 with the MiniMax Imager (Molecular Devices, Sunnyvale, CA). Angiogenesis was analyzed with ImageJ software using the angiogenesis analyzer. The primers used to quantify HOXA5 were (CTGGTTCCAAAACAGGAGGA, ACGAGAACAGGGCTTCTTCA). Expression was normalized to GAPDH.

### mRNA-sequencing

HUVECs were cultured to 80% confluency and treated with 7.5ug/ml of MSC exosomes. After 36 hours RNA was collected with the RNeasy kit (Qiagen) and analyzed by mRNA-sequencing using HiSeq. 2000 100PE. Non-treated and treated HUVECs were each analyzed in triplicate (n = 3). Paired-end reads were aligned to the human genome version 38 using STAR version 2.5.3a. The resultant SAM files were converted to BAM in Galaxy and then analyzed using htseq-count, followed by DEseq. 2 to reveal differentially expressed genes following treatment with MSC exosomes.

### Cardiomyocyte proliferation and apoptosis

Cardiomyocytes were isolated from murine hearts derived from 18 day old ICR mice using the Pierce cardiomyocyte isolation kit (Thermofisher) and cultured according to the manufacturer’s protocol^4^. All methods and experiments were performed in accordance with the U.S National Institute of Health Guide for Care and Use of Laboratory Animals. Experiments were approved by the Institutional Animal Care and Use Committee of the University at Buffalo. Cardiomyocytes were plated onto cell culture dishes coated with poly-L-lysine (2 µg/cm^2^). 72 h after plating, cardiomyocytes were incubated with 0.5 μg and 2 μg of unmodified exosomes and exosomes loaded with miRNAs for 24 h (n = 9). Cardiomyocyte apoptosis was detected using the Click-iT™ Plus TUNEL Assay for *In Situ* Apoptosis Detection Alexa Fluor™ 488 dye (Thermofisher)^4^. Cardiomyocyte proliferation was detected using the Click-iT™ EdU imaging kit with Alexa Fluor 488 dye. Cardiomyocytes were incubated with 5-ethynyl-29-deoxyuridine (EdU) for 24 h before analysis was performed (n = 3). Nuclei were stained with Hoechst 33342. Anti-Troponin T (Thermofisher, Cat#: MA512960) was used to stain cardiomyocytes. 72 h after plating ~95% of the cells stained positive for Troponin I (Abcam, ab47003) and Troponin T. ImageJ was used to count the number of Hoechst stained nuclei and EdU stained nuclei/or TUNEL stained nuclei. Cardiomyocyte proliferation was calculated as: ((# EdU nuclei/# Hoechst nuclei)*100). Cardiomyocyte apoptosis was calculated as: ((# TUNEL nuclei/# Hoechst nuclei)*100). Data are expressed as mean ± sd. The primers used to quantify Crim1 were (CAAAGGTAGGGCCTGAATGA, GGCTTCCTCACTGCCTACTG). Expression was normalized to GAPDH.

### Apoptosis Induction Assay

Plated cardiomyocytes as described above were treated with 2ug/ml of unmodified MSC exosomes and 2 ug/ml of miR-199a-3p loaded MSC exosomes after 96 hours in culture. At 44 hours post exosome treatment cardiomyocytes were treated with 1.6 mM of hydrogen peroxide. After 4 hours RNA was collected with the RNeasy kit (Qiagen) and cDNA libraries were generated with the ProtoScript II kit (NEB). Apoptosis and oxidative stress markers were quantified by qPCR. The primers used were: (p53 AGAGACCGCCGTACAGAAGA, CTGTAGCATGGGCATCCTTT), (BAX TGCAGAGGATGATTGCTGAC, GATCAGCTCGGGCACTTTAG), (CASP9 TGCCCTTGCCTCTGAGTAGT, AACAAAGAAACGCCCACAAC), (NFkB CTGACCTGAGCCTTCTGGAC, GCAGGCTATTGCTCATCACA). Expression was normalized to GAPDH and beta actin.

### Fibrosis assay

Primary human cardiac fibroblasts were obtained from ScienCell (Cat# 6330). Cells with passage numbers P3-P7 were used. Cells were plated onto a 24 well plate, cultured in fibroblast medium-2 (Cat.#2331) and grown to 80% confluency. Cells were then serum starved for 12 hours in 500 μl of DMEM with or without MSC exosomes ranging from 0.1 μg to 8.0 μg. After 12 hours, cells were stimulated for fibrosis by supplementing with an additional 500 μl of complete media containing TGF-β (Peprotech) to a final concentration of 10ng/ml. To assess collagen production cells were lysed after 48 hours with 10% SDS and RNA was extracted according to the Trizol protocol. cDNA was synthesized with the SuperScript II First Strand Synthesis Kit (Invitrogen) and analyzed for expression of collagen I (primer sequences: FP: GGGCAAGACAGTGATTGAATA and RP: ACGTCGAAGCCGAATTCCT) and GAPDH (ThermoFisher, RevertAid) by RT-PCR using SYBR Mastermix (Quanta) on a Stratagene Mx 3000 P.

### Enrichment of miRNAs into exosomes

For exosomal enrichment, miRNAs were electroporated into MSCs cultured in exosome-depleted media using the Neon Electroporation System (Thermo Fisher Scientific). 1.2 × 10^5^ MSCs were electroporated with 3 µg (low dose) and 15 µg (high dose) of miRNAs using the following settings: 990 volts, 40 millisecond pulse, one pulse. miR-199a-3p, sense: (5′-ACAGUAGUCUGCACAUUGGUUA-3′) antisense: (5′-UAACCAAUGUGCAGACUACUGU-3′) and miR-130 sense: (5′-CAGUGCAAUGUUAAAAGGGCAU-3′) antisense: (5′-AUGCCCUUUUAACAUUGCACUG-3′) were used for electroporation. Cells were washed thoroughly to remove excess RNA. Exosomes were collected 48 h after electroporation and analyzed for miR-130a-3p and miR-199a content by RT-PCR using qScript microRNA cDNA Synthesis Kit (Quanta) and SYBR mastermix (Quanta) on a Stratagene Mx 3000 P.

### Exosomal uptake and flow cytometry

To assess exosome uptake into cardiomyocytes and HUVECs, cells were incubated with Bodipy-TR-labeled exosomes for 0.5 h, 1 h, 2 h, 4 h, and 24 h and washed thoroughly using PBS. Cellular binding and/or uptake were determined by flow cytometry (MACSQuant; Miltenyi Biotech, Woking, UK).

### Statistical analyses

GraphPad Prism 7 was used to perform all statistical analyses. One-way Anova followed by Tukey’s post test was used for the analyses of multiple groups. Statistically significant differences were marked with asterisks (*p < 0.05, **p < 0.01, ***p < 0.001).

## Electronic supplementary material

Supplementary data

Supplementary File_Excel table 1

Supplementary File_Excel table 2
