# Supplementary material for: The microRNA regulatory landscape of MSC-derived exosomes: a systems view
Source: Sci Rep. 2018 Jan 23;8:1419. doi: 10.1038/s41598-018-19581-x (PMC5780426; doi:10.1038/s41598-018-19581-x)
Supplement: Supplementary file 1 — Supplementary data [file 41598_2018_19581_MOESM1_ESM.pdf]

The microRNA regulatory landscape of MSC-derived exosomes: a systems view

Scott W. Ferguson, Jinli Wang, Christine J. Lee, Maixian Liu, Sriram Neelamegham, John M. Canty, Juliane Nguyen

Supplementary Information

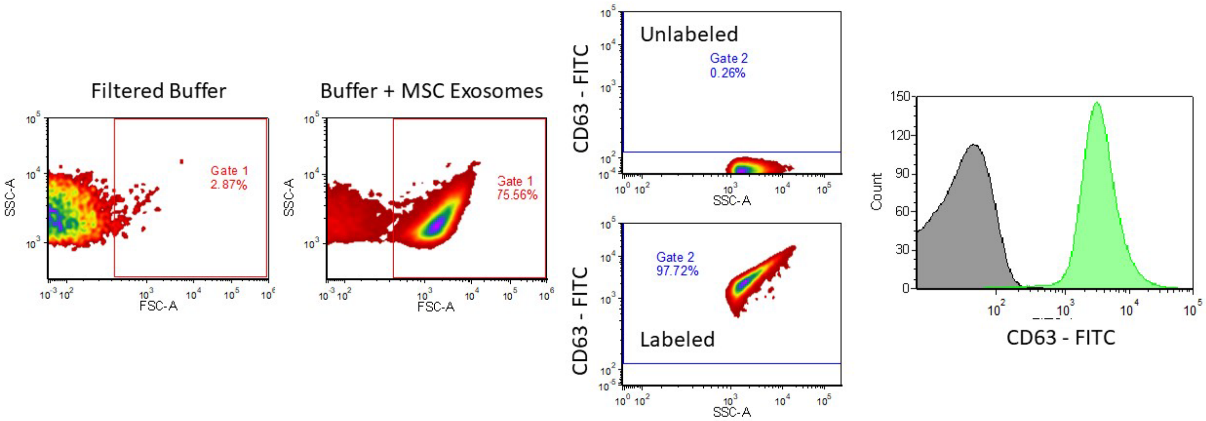

Supplementary Figure 1: Analysis of exosomes for CD63 expression by flow cytometry. 50µg MSC exosomes in 100µl of filtered PBS were incubated with 5µl of anti-CD63-FITC antibody (Miltenyi) at 4°C for 1 hour. Samples were analyzed on the MacsQuant flow cytometer using manufacturer suggested settings for exosomes.

A

	Number of genes targeted	Circulatory System Development	Growth
**miR-23a-3p**	1069	104	176
**miR-424-5p**	956	94	195
**miR-144-3p**	780	85	136
**miR-130a-3p**	742	99	180
**miR-145-5p**	660	66	117
**miR-29b-3p**	635	65	129
**miR-29a-3p**	629	65	127
**miR-25-3p**	604	75	120
**miR-221-5p**	456	55	107
**miR-21-5p**	439	61	104
**miR-125b-5p**	436	43	93
**miR-22-3p**	422	44	95
**miR-199a-3p**	416	53	73
**Let-7a-5p**	174	23	29
**Let-7i-5p**	168	21	28
**Let-7b-5p**	166	22	28
**miR-630**	74	38	66
**miR-191-5p**	72	13	19
**miR-1246**	61	14	8
**miR-451a**	43	10	13
**miR-100-5p**	33	9	6

B

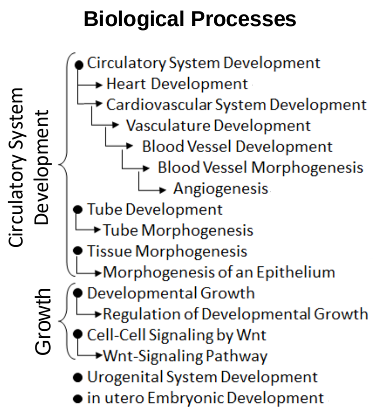

C

	Number of Genes Targeted	GnRH receptor	Angio genesis	Integrin signaling	CCKR Signaling map	Wnt signaling	TGF-beta signaling	PDGF signaling	EGF signaling	PI3K signaling	P53
**miR-23a-3p**	1069	21	10	12	18	14	4	9	10	5	5
**miR-424-5p**	956	28	13	13	15	16	8	12	9	6	3
**miR-144-3p**	780	19	6	10	9	22	5	10	9	5	4
**miR-130a-3p**	742	20	14	8	14	18	10	12	9	3	5
**miR-145-5p**	660	16	9	12	11	10	9	10	4	1	0
**miR-29b-3p**	635	11	10	25	11	15	4	5	9	6	6
**miR-29a-3p**	629	11	8	25	11	15	4	6	8	5	6
**miR-25-3p**	604	10	3	11	10	10	5	3	4	2	2
**miR-221-5p**	456	11	8	5	6	16	6	4	4	1	2
**miR-21-5p**	439	10	4	6	6	5	10	5	6	1	0
**miR-125b-5p**	436	10	4	3	9	5	2	6	7	1	1
**miR-22-3p**	422	12	6	2	8	5	7	4	6	4	6
**miR-199a-3p**	416	13	7	9	10	9	4	3	8	2	0
**Let-7a-5p**	174	3	2	5	0	3	4	2	1	1	1
**Let-7i-5p**	168	3	1	6	0	3	4	3	1	1	1
**Let-7b-5p**	166	3	1	5	0	3	4	3	1	1	1
**miR-630**	74	13	6	2	7	7	4	3	4	1	1
**miR-191-5p**	72	4	3	2	1	1	2	0	0	0	1
**miR-1246**	61	2	1	0	1	3	1	0	1	0	0
**miR-451a**	43	1	0	0	1	1	1	0	1	0	1
**miR-100-5p**	33	1	0	2	0	2	0	0	0	0	0

Supplementary Figure 2. (A) Number of genes targeted by the top 23 miRNAs found in MSC exosomes categorized into total number of genes targeted, genes related to circulatory system development, and genes related to growth. (**B**) Biological processes that are categorized into circulatory system development and growth, (**C**) Number of genes targeted categorized into total number of genes targeted and enriched pathways.

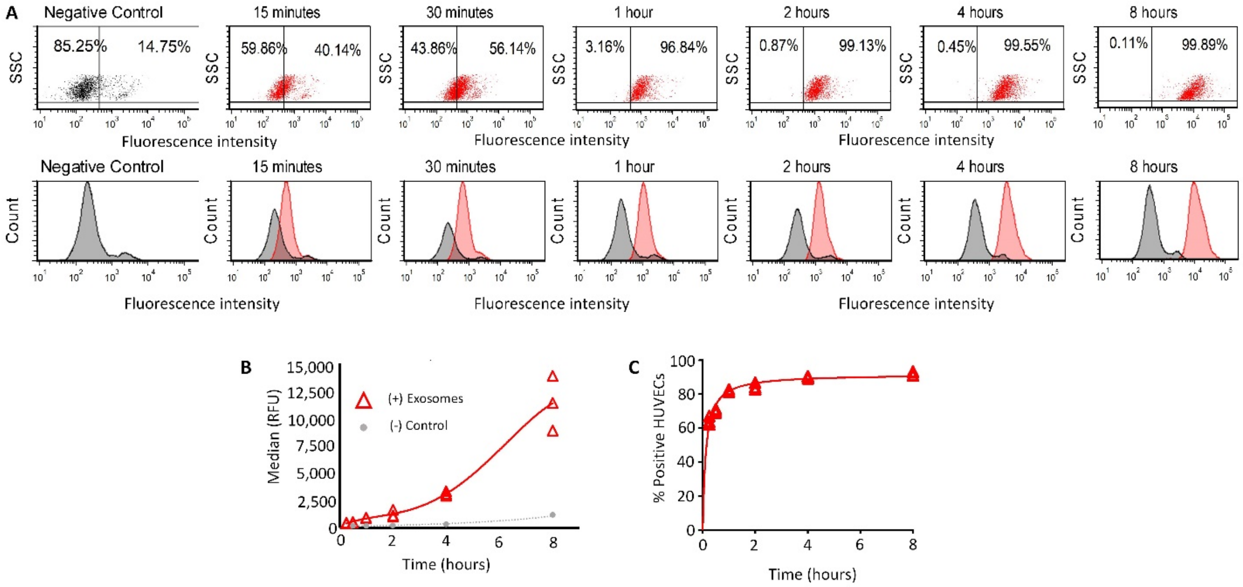

Supplementary Figure 3. Cellular binding and uptake of exosomes into HUVECs as a function of time (0.5h, 1h, 2h, 4h, 6h, and 8h).

To assess uptake kinetics of exosomes into endothelial cells, HUVECs were incubated with Bodipy-TR-labeled exosomes for up to eight hours. Exosome uptake increased with increasing incubation time. A 12-fold increase in exosomal binding or uptake was observed at 8 h of incubation compared to 1h and 2h of incubation. At 30 minutes of incubation, only 56% of cells showed exosome binding and or uptake, which increased to ~95% after 1 h and further to 99.1% and 99.9% after 2 to 8 hours of incubation. Further, the percentage of exosome-positive HUVECs increased over time (~1,000 MFI at 1 h vs. ~12,500 MFI at 8 h).

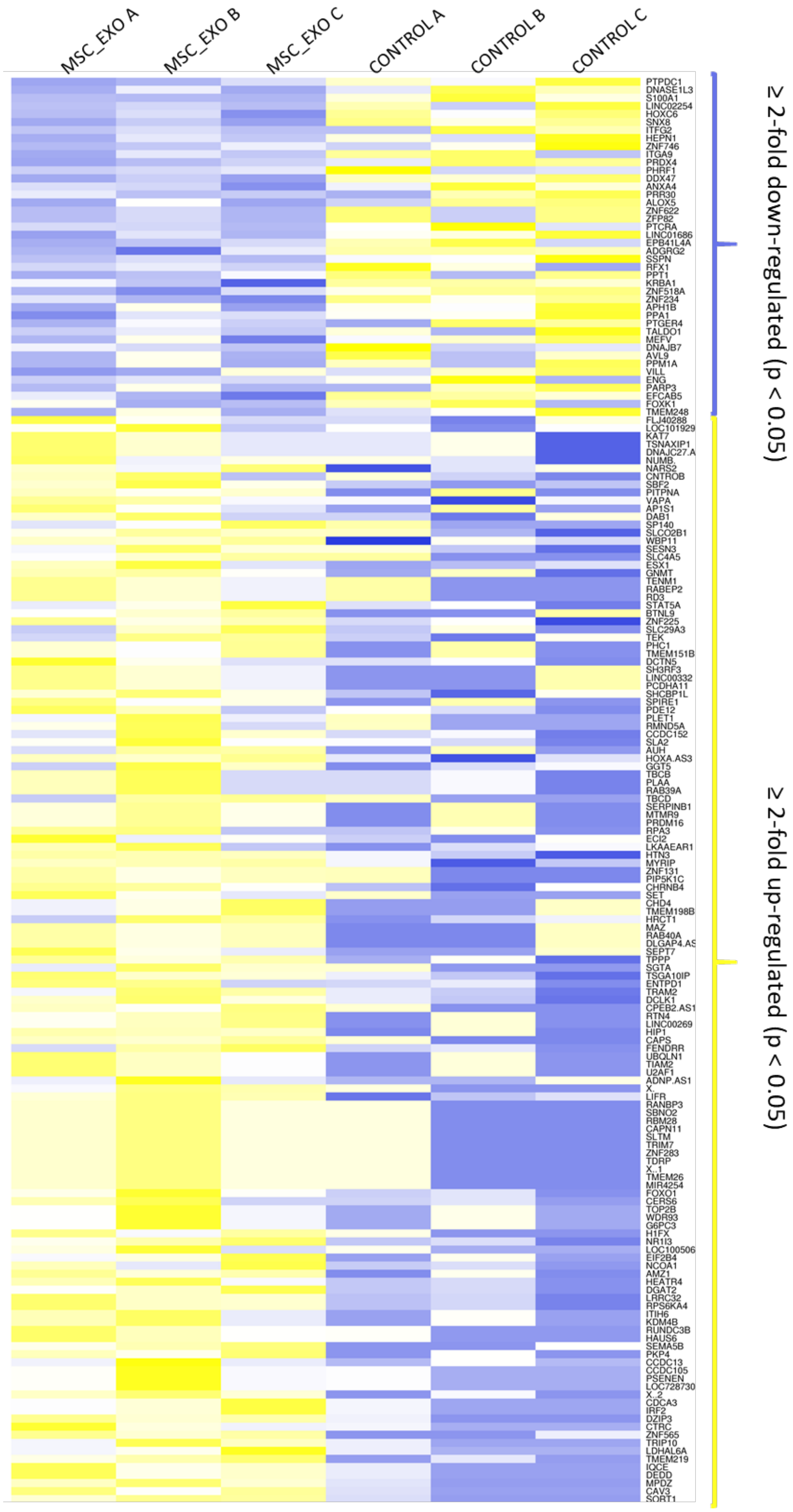

Supplementary Figure 4. Heatmap illustrating all genes in HUVECs that were up- or down-regulated (≥ 2-fold) as a result of exposure to MSC exosomes achieving statistical significance at $p < 0.05$. RNA reads were aligned using STAR version 2.5.3a. The resultant SAM files were converted to BAM in Galaxy and then analyzed using htseq-count, followed by DEseq2 which provides fold changes as well as statistical significance.

Unique Gene Targets of miR-199a-3p	Other Non-Unique Gene Targets of miR-199a-3p		
CASP9	CRIM1	ERBB4	NF1
CABLES1	NET1	ATRX	DNMT3A
LKB1	GRHL3	PAK1	CNOT7
NEUROD1	PRKACB	MECP2	CDC42
RB1	GREM1	CDC14A	ITGA6
DEPDC1B	NR4A3	ARHGEF12	VANGL1
KIAA0141	MAP3K1	DCBLD2	PIK3R1
ITGA3	ANGPT1	UFM1	PTPN3
MAP3K5	PRKCE	YWHAE	PTPRJ
CHMP5	CITED2	SEMA3A	YAP1
ARHGEF2	GPR37	MEIS2	AREL1
SLC46A2	DDIT4	SH3GLB1	PTPRU
PRPF40A	NTRK2	MAPRE1	SGK3
SERPINE2	PLCB1	RARB	TAOK1
DLX5	FOS	RUNX1	GOLGA4
ARG2	CXCL12	FN1	TRIM36
ATP6V1C2	CPEB4	GDF9	CYP1B1
SRA1	MAP3K4	CARD6	BCL2L14
PAK4	FBXW11	CHL1	
CELSR2	UBQLN1	PDE8A	
USP8	SLC39A10	PAWR	
HGF	HNF1B	ARHGEF3	

Supplementary Table 3: Gene targets of the miRNAs were compared to the GO Terms: Regulation of Cell Death (GO:0010941), Growth (GO:0040007), Cell Cycle (GO:0051726) and Wnt-signaling Pathway (GO:0016055). Twenty two genes curated in regulation of cell death, growth, cell cycle, or Wnt-signaling pathway were unique to miR-199a-3p.

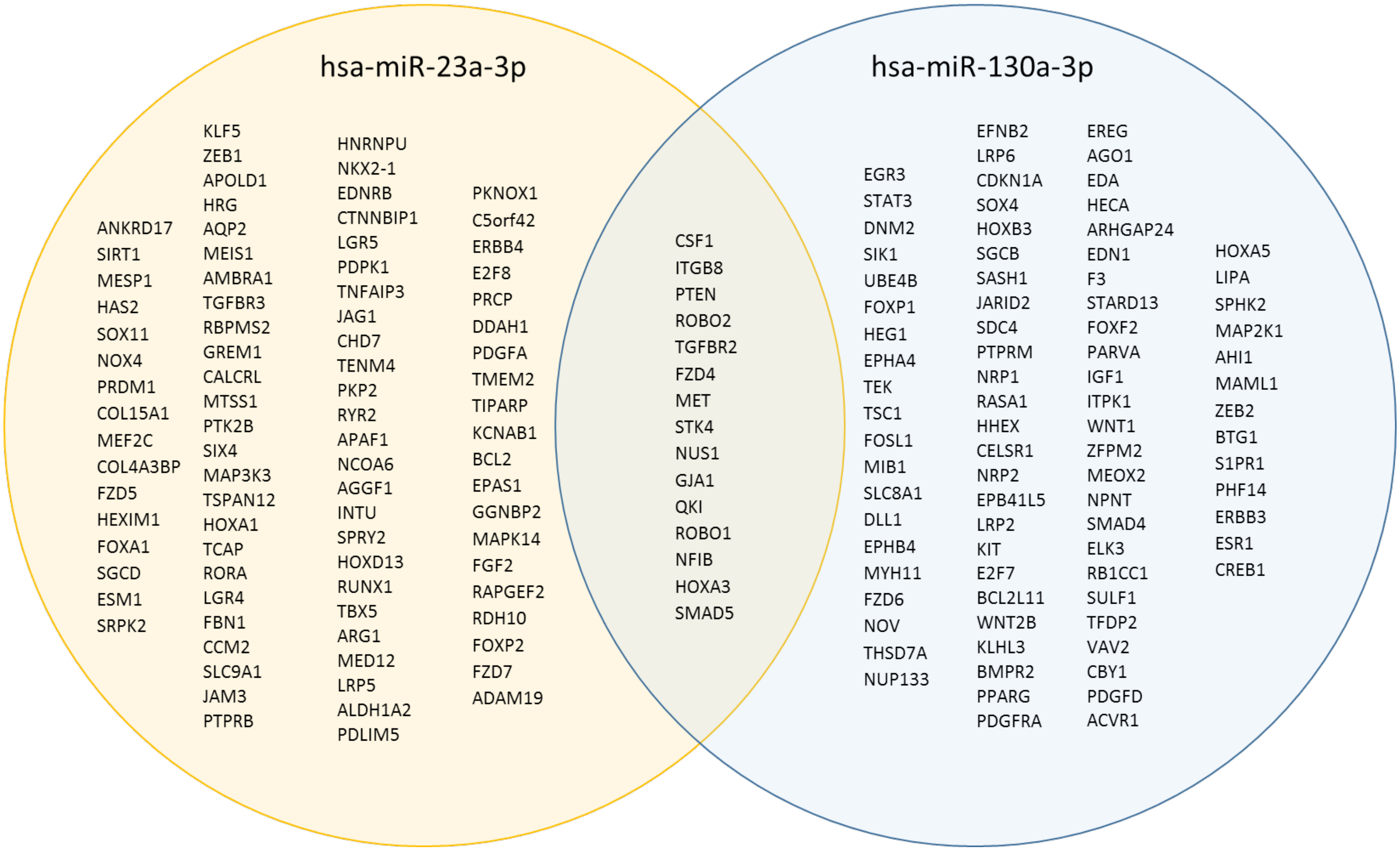

Supplementary Figure 5. The Venn diagram depicts the 104 and 99 genes related to “Circulatory System Development” (Supplementary Figure 2A) for hsa-miR-23a-3p and hsa-miR-130a-3p.

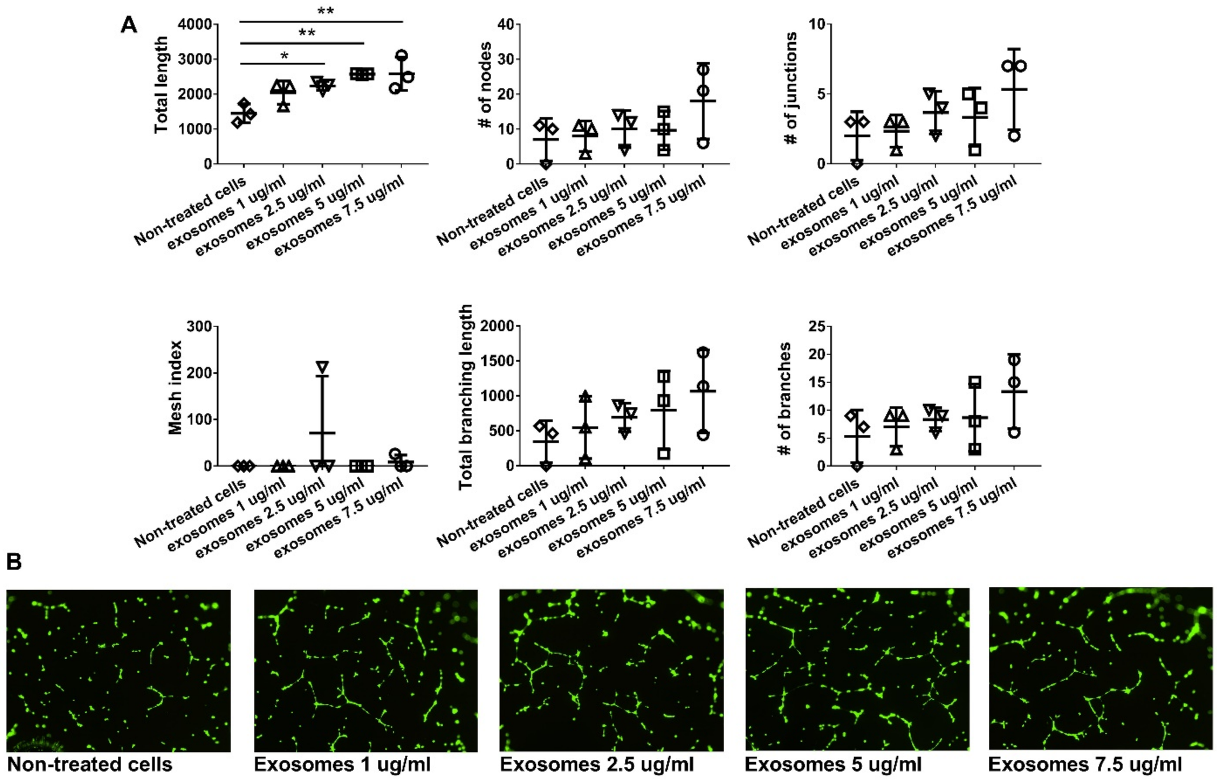

Supplementary Figure 6. (A) Effect of unmodified MSC exosomes on angiogenesis in HUVECs as analyzed using the ImageJ angiogenesis analyzer. (B) Calcein stained HUVECs treated with increasing concentrations of exosomes. Data are mean ± sd, n=3, by one-way ANOVA with Tukey post-test (*p<0.05, **p<0.01).
